# Prognostic Significance of Simple Scoring Systems in the Prediction of Diffuse Peritonitis Morbidity and Mortality

**DOI:** 10.3390/life12040487

**Published:** 2022-03-28

**Authors:** Petr Špička, Josef Chudáček, Tomáš Řezáč, Lubomír Starý, Rostislav Horáček, Dušan Klos

**Affiliations:** 1 First Department of Surgery, University Hospital Olomouc, 779 00 Olomouc, Czech Republic; tomas.rezac@fnol.cz (T.Ř.); lubomir.stary@fnol.cz (L.S.); dusan.klos@fnol.cz (D.K.); 2 Department of Anesthesiology, Resuscitation and Intensive Care, University Hospital Olomouc, 779 00 Olomouc, Czech Republic; rostislav.horacek@fnol.cz

**Keywords:** peritonitis, ASA, MPI, qSOFA, ECOG, NPWT

## Abstract

Introduction: Diffuse peritonitis is a serious disease. It is often addressed within urgent management of an unstable patient in shock. The therapy consists of treatment of the source of peritonitis, decontamination of the abdominal cavity, stabilization of the patient and comprehensive resuscitation care in an intensive care unit. A number of scoring systems to determine patient prognosis are available, but most of them require complex input data, making their practical application a substantial problem. Objective: Our aim was to assess simple scoring systems within a cohort, evaluate the level of mortality, morbidity, and duration of hospital stay, followed by a comparison of the acquired data with the literature and determination of an easily implementable scoring system for use in clinical practice. Material and Methods: We evaluated a group of patients with diffuse peritonitis who underwent surgery in the 2015–2019 period. Medical history, surgical findings, and paraclinical examinations were used as the input for four scoring systems commonly used in practice—MPI, qSOFA, ECOG, and ASA. We compared the results between the systems and with the literature. Results: Our cohort included 274 patients diagnosed with diffuse peritonitis. Mortality was 22.6%, morbidity 73.4%, with a 25.2 day average duration of hospital stay. Mortality and morbidity increased with rising MPI and qSOFA, well-established scoring systems, but also with rising ASA and ECOG, similarly to MPI and qSOFA. Conclusions: The utilized scoring systems correlated well with the severity of the condition and with predicted mortality and morbidity as reported in the literature. Simple scoring systems primarily used in other indications (i.e., ASA and ECOG) have a similar predictive value in our cohort as commonly used systems (MPI, qSOFA). We recommend them in routine clinical practice due to their simplicity.

## 1. Introduction

Despite improving diagnostic and treatment options, diffuse peritonitis is a disease with high morbidity and mortality. Patients admitted to hospital with diffuse peritonitis are often in a state of shock. These patients often suffer from a number of comorbidities, are of higher age, and utilize polypharmacotherapy. The management is primarily surgical and is based on the treatment of the cause of peritonitis, decontamination of the abdominal cavity, lavage, drainage, and is then usually continued at an intensive care unit with targeted antibiotic therapy and comprehensive intensive care.

Acute peritonitis is defined as an inflammation of the peritoneum—the serous membrane lining the abdominal cavity, both its wall—the parietal peritoneum—and the organs of the abdominal cavity—the visceral peritoneum.

Based on the clinical extent, peritonitis can be classed as localized (limited to a certain part of the abdominal cavity) or diffuse (where the inflammation spreads to other parts of the abdominal cavity). The clinical classification is important mainly for further treatment, since while signs of diffuse peritonitis in clinical examination are usually considered to be a clear indication for surgical management, localized peritonitis can be further monitored to select appropriate therapy [1]. Surgical therapy is usually utilized in secondary peritonitis, although other types of peritonitis should also be mentioned. Primary peritonitis is defined as a spontaneous bacterial colonization of the peritoneal cavity, typically in patients with ascites in liver cirrhosis—an ideal breeding ground for bacterial contamination. Peritonitis is referred to as secondary if the peritoneum is in direct contact with an infectious noxa, most commonly after perforation of a hollow organ in the abdominal cavity. This is why secondary peritonitis is usually polymicrobial while primary peritonitis is frequently oligobacterial or monobacterial. Tertiary peritonitis is the next type, defined as “peritonitis that lasts longer than 48 h in adequate treatment of secondary peritonitis” [2]. This type of peritonitis is usually diagnosed based on clinical signs—prolonged sepsis in a patient with adequately managed secondary peritonitis, often requiring additional unplanned surgical revisions to control intra-abdominal sepsis.

In addition to this typical classification of peritonitis, it is convenient to classify secondary peritonitis with respect to the nature of the effusion, especially for further clinical assessments. Here, we distinguish between serous, fibrinous, hemorrhagic, chemical, purulent, stercoral, biliary, or urinary peritonitis.

Although a number of other peritonitis classification systems exist, we considered the above systems adequate from the surgical point of view, especially for the further treatment management and evaluation of its effect.

Peritonitis is most often caused by exposure of the peritoneum to infectious noxa when perforating a hollow organ of the abdominal cavity, for example, by foreign bodies, bile during gallbladder or intrahepatic or extrahepatic bile duct perforation, gastric acids during gastric or duodenal ulcer perforation, urine during bladder perforation, etc. In women, peritonitis may occur with ovarian cyst rupture or fallopian tube infections. Regarding the clinical symptoms, their range is very wide, from the inconspicuous development of abdominal pain in the beginning, leading to severe septic shock with a direct threat to the patient’s life.

The diagnosis of peritonitis is usually based on typical clinical features. Laboratory and paraclinical examinations are standard, with the help of which the diagnosis of the disease is usually quite reliable. Recently, peritoneal lavage or paracentesis have rarely been used to verify the pathological process of the peritoneum and abdominal cavity. Additional laboratory examination is necessary in the algorithm of examination methods if diffuse peritonitis is suspected—according to department practice—and if there is no significant time lag before the therapeutic intervention, imaging examinations are suitable—at least an X-ray examination of the abdomen, ultrasound examination of the abdomen, and possibly CT examination, which can significantly contribute to the diagnosis of diffuse peritonitis.

Surgical treatment is essential. It is always primarily necessary to treat the cause and then to perform a perfect decontamination of the abdominal cavity. Two further approaches to the management are possible—temporary abdominal closure with elective surgical control of the abdominal cavity in 24–72 h, most often via NPWT or non-woven fabric (“COM”), or primary closure with abdominal drainage and possible postoperative lavage [3]. Continuous postoperative abdominal lavage used to be a relatively common procedure for the management of diffuse peritonitis, especially in German-speaking countries in advanced stercoral peritonitis. However, the effect of lavage has been questioned over time [4,5]. Vacuum therapy (NPWT, VAC) has been increasingly used in more severe forms of diffuse peritonitis—it seems to be more beneficial in many respects than simple perioperative lavage [6]. There are currently a number of prospective studies that are seeking to find the best surgical approach to the treatment of patients with diffuse peritonitis, in order to select the optimal procedure with the least number of complications, and the lowest mortality and morbidity with the best benefit for the patient. Continuous postoperative lavage has a long history in our department. It is still used today, although to a much lesser extent. Diffuse peritonitis is often management with primary closure of the abdominal cavity and abdominal drainage. NPWT therapy has also been utilized in these conditions in the last few years at our department, even in an ongoing prospective randomized study to evaluate the effectiveness of these methods.

Diffuse peritonitis is still associated with high medical and social severity, and a mortality percentage that remains in double digits. There are a number of scoring systems to estimate the risk of morbidity and mortality in diffuse peritonitis. However, these systems are relatively complicated, requiring complex input data not readily available to the average surgeon in clinical practice. This study was based on the retrospective evaluation of data obtained in the 2015–2019 period within diffuse peritonitis patient surgeries at our department. ASA, ECOG, MPI at the time of surgery, and qSOFA scores were determined. Predicted morbidity and mortality with these scoring systems were then compared, both between individual scoring systems and with the available global literature.

## 2. Material and Method

The study was initiated in January 2015 by the determination of data to be monitored in patients and of the study group. Data collection was completed in December 2019. All patients with a perioperatively confirmed diagnosis of diffuse peritonitis were included in the study. Peritonitis cases were then divided into four groups according to the nature of the effusion: (1) Serous, chemical and other peritonitis (fibrinous, hemorrhagic, urinary); (2) biliary peritonitis; (3) purulent peritonitis; and (4) stercoral peritonitis.

The patients were operated on for symptoms of acute abdomen. First, a thorough toilette of the abdomen was performed and the primary cause of diffuse peritonitis was treated, usually by resection of the affected organ. Subsequently, a method for the treatment of diffuse peritonitis was chosen. In 66 patients, vacuum therapy (NPWT, V.A.C.) was chosen as the primary treatment, and 18 patients underwent surgery with implantation of non-woven textile under the fascia as a temporary abdominal closure as part of the planned re-laparotomy. For both procedures, another surgery was planned in 24–48 h. Postoperative continuous lavage was applied to 84 patients (usually four drains in each quadrant of the abdominal cavity followed by continuous lavage—250 mL of antiseptic solution was instilled in each drain for two hours; for the next two hours, the drains were left without instillation, usually this rotated for 24–48 h) and in 106 patients, the primary abdominal wall closure with abdominal drainage was chosen as the definitive procedure. These two procedures, unlike the previous ones, were not associated with another planned laparotomy and were therefore assessed as definitive. Therefore, if another surgery in this group was needed, it was evaluated as a complication according to the Clavien-Dindo classification at least as IIIb.

Thirty-day mortality, morbidity according to Clavien-Dindo classification II, and the duration of hospitalization in the monitored group were evaluated. The assessment of morbidity was deliberately strict (i.e., grade II according to the CD classification) to obtain the most critical information for patients and therapy. The following variables were considered significant (CD II and more) for postoperative morbidity: severe infectious complications including intra-abdominal infections, heart rhythm disorders and substantial hypertension, neurological complications, bleeding including the need for blood transfusions, the need for endoscopic, imaging or surgical intervention, forced surgical revisions, and failure of one or more organs.

MPI (Mannheim Peritonitis Index) and preoperative ASA score were determined from the available data. The preoperative ECOG status was evaluated and a qSOFA scoring system was utilized.

MPI is one of the most widely used scoring systems in patients with diffuse peritonitis, especially in terms of mortality prediction. Evaluated parameters are: age, sex, presence of organ failure, malignancy surgery, origin of sepsis outside the colon, duration of peritonitis, extent of peritonitis, and nature of exudate. The disadvantage is the relatively large amount of data obtained. The advantage is the large informative value of the system. The ASA score is a well-established system used mainly by anesthesiologists, which assesses the patient’s physical status on the basis of mild and severe comorbidities present and, in contrast, is a very simple and fast system. ECOG (Eastern Cooperative Oncology Group) status describes a patient’s level of functioning in terms of their ability to care for themselves, daily activity, and physical ability (walking, working, etc.). This patient physical condition assessment system is primarily intended for cancer patients. qSOFA is a simplified form of the established SOFA scoring system used in intensive care units, especially in Anglo-Saxon countries, as a rapid clinical scoring system, helping to identify septic patients at high risk of morbidity and mortality. The primary outcome in the case of SOFA is mortality during hospitalization, and the secondary outcome is the stay in the ICU ≥3 days. qSOFA is also a very simple system. The parameters that were evaluated are the degree of consciousness, respiratory rate, and systolic blood pressure.

The results were then compared between individual scoring systems and with the literature. Quantitative variables were presented using means and standard deviations (SD), minimum and maximum values, and medians. Shapiro–Wilk normality tests showed that quantitative quantities did not have normal distribution. Therefore, non-parametric methods were used for data processing. Mann–Whitney U-test was used to compare two independent samples; Kruskal–Wallis test with subsequent Dunn’s post hoc tests was used to compare multiple independent groups. Qualitative data were described using absolute and relative frequencies. The differences were verified using Fisher’s exact test. All tests were performed at the statistical significance level of 0.05. Results with a *p*-value less than 0.05 were considered statistically significant. The data were analyzed using IBM SPSS Statistics for Windows, Version 23.0. Armonk, NY, USA: IBM Corp. and TIBCO STATISTICA version 13.4.0.14. 

## 3. Results

**Figure 1:**
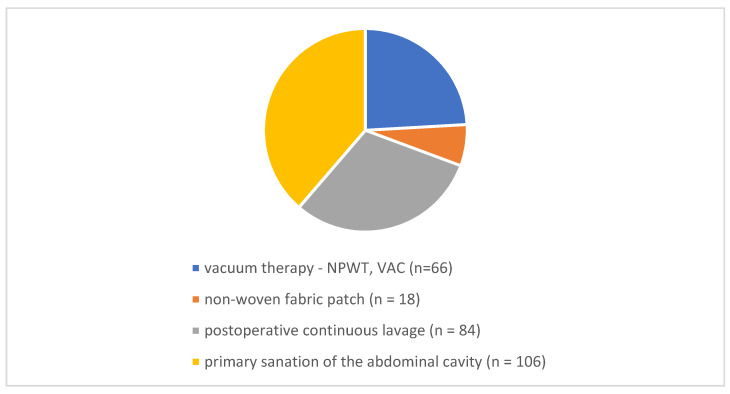
Type of surgery and patient number in the treatment of diffuse peritonitis.

A total of 274 patients with diffuse peritonitis were operated on at our department between 1/2015 and 12/2019. Sixty-six patients were treated with vacuum therapy (NPWT, VAC); a non-woven fabric patch (“COM”) was utilized in 18 patients as part of a planned relaparotomy; 84 patients underwent postoperative continuous lavage; and 106 patients underwent primary abdominal wall closure as the definitive procedure (Figure 1). 

**Figure 2:**
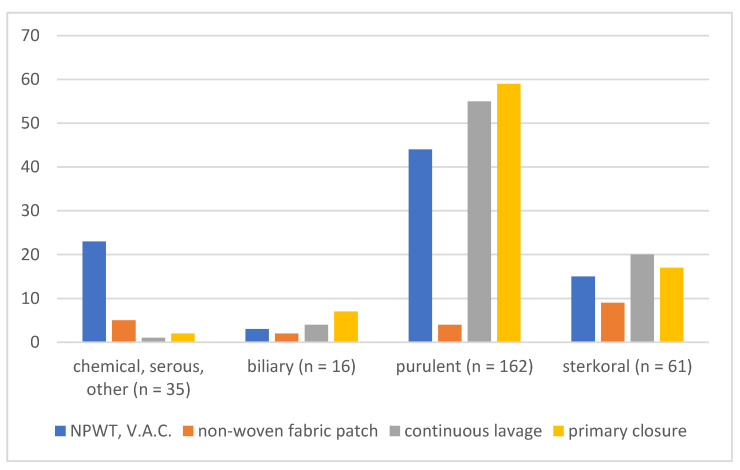
Individual types of peritonitis according to the nature of the effusion and method of treatment.

Serous, chemical, and other peritonitis (1) was present in 35 patients, biliary peritonitis (2) in 16 patients, purulent peritonitis (3) in 162 patients, and stercoral peritonitis (4) in 61 patients (Figure 2). 

There were 56.2% men in our group and 43.8% women, meaning the difference between the two groups was statistically non-significant (*p* = 0.670). The age distribution was symmetrical, and the average age of the patients was 61.9 ± 16.6 years. The total 30-day mortality in our group was 22.6%, without statistical significance between the years 2015–2019 (*p* = 0.820). The total morbidity in our group was 73.4%, again without statistical significance between individual years (*p* = 0.166). The duration of hospitalization was 25.2 ± 22.5 days (*p* = 0.651).

First, we related mortality and morbidity to age. On data processing, we found a clear dependence of both mortality and the presence of postoperative complications on age—both values increased significantly with increasing age (*p*<0.0001)—Table 1.

Furthermore, patients were divided into groups according to individual scoring systems. Established systems for the prediction of mortality and morbidity in diffuse peritonitis—MPI (Mannheim Peritonitis Index), qSOFA was used in the first place. Afterward, simple scoring systems were used, primarily to assess the overall condition of patients. The ASA system used for over 60 years was chosen due to its simplicity and wide distribution. ECOG performance status, particularly widely used in oncological patients, was also utilized for the same reasons. We subsequently calculated the total mortality and morbidity according to individual scoring systems (Table 2).

MPI values were used to divide patients into three groups with the cut-off values set at 20 and 29, respectively [3,7]. The total 30-day mortality in the MPI 0–20 group was 8.6%, 9% in the MPI 21–29 group, and 44.6% in the MPI 30 and over group (*p* < 0.0001). Morbidity was compared using the same procedure—the total morbidity in the individual MPI groups was 43.1%, 73%, and 90.5%, respectively (*p* < 0.0001). 

Mortality and morbidity were also evaluated utilizing other scoring systems. A very good correlation of results was found. A slight decrease in morbidity was only observed in advanced findings (severe peritonitis with qSOFA 3 or a severely polymorbid patient (ASA IV, ECOG 4), probably due to the smaller size of the group. A slight decrease in mortality was recorded in ECOG 4 compared to ECOG 3 (Table 2 and Table 3), probably for the same reason.

## 4. Discussion

Diffuse peritonitis is a disease with high occurring mortality also shown in recent data. Tolonen et al. reported an overall 30-day mortality of 14.5%, with mortality fluctuating extremely based on the presence or absence of severe comorbidities [8]. It is evident that the patient’s overall condition is an essential aspect of further prognosis. There are a number of scoring systems, but the vast majority are still not readily usable for routine surgical practice. Mannheim Peritonitis Index (MPI), POSSUM, APACHE II, and the Peritonitis Severity Score (PSS) are considered to be the most effective [9,10,11,12,13]. MPI, in particular, seems to be a valid scoring system capable of predicting the fate of patients with diffuse peritonitis rather well. Different mortality levels were reported in individual groups based on MPI values. Mortality rises with increasing MPI, which was also demonstrated in our study [7,14]. 

Another scoring system used and evaluated in our cohort was qSOFA—a simplified form of the established SOFA scoring system used in intensive care units, especially in Anglo-Saxon countries. It is a quick clinical scoring system intended to identify septic patients at high risk of morbidity and mortality. The primary outcome of this system is in-hospital mortality, followed by ICU hospitalization for ≥3 days [15]. A 3–14-fold increase in in-hospital mortality was observed in this study in patients with qSOFA ≥2 at the time of admission. The mortality increase in qSOFA ≥2 was lower in our cohort than in the literature. However, even our results correlated well with the literature data, where increasing qSOFA is associated with increasing mortality.

Of much interest is the recent work of Petersen et al., who examined the prediction of survival based on the novel multi-domain peritonitis predictive model (MPPM) in 1351 patients managed with open abdomen technique between 1998 and 2018. This system consists of several variables combining demographic, physiological, and surgical data. Based on the model, skin closure was found to be the best mortality predictor in these patients, followed by the scoring systems SAPS (Simplified Acute Severity Score) II and MPI [16]. We went the opposite way, attempting to find the simplest possible predictor of survival and thus identify the degree of peritonitis severity based on the smallest possible input dataset, thereby enabling the prediction to be made as early as the preoperative or perioperative phase. It is noteworthy that although our cohort of 274 patients was recruited between 2015 and 2019, the yearly patient numbers were similar to those in Petersen’s work. Furthermore, the cohort was acquired during the last 5–10 years when the new surgical procedures such as NPWT had already been fully standardized.

An even simpler scoring system was thus being considered due to the underlying disease. This system could be used for the same purpose, although primarily intended to evaluate other variables. Therefore, the standard ASA scoring system used in surgical practice was chosen together with the ECOG Performance Status, a tool to evaluate the overall condition in oncology patients.

The ASA score seems to be of particular interest for the prediction of mortality and morbidity, which is also based on the available literature [17]. Farrow et al. reported the following mortality of surgical patients in individual ASA groups: 0–0.3% in ASA I, 0.3–1.4% in ASA II, 1.8–4.5% in ASA III, 7.8–25.9% in ASA IV, and 9.4–57.8% in ASA V [18]. Similarly, ASA scores have also been used in the assessment of surgical morbidity [19]. In one of the few articles available on ASA scores and diffuse peritonitis, Casal Núñez assessed the morbidity and mortality in patients undergoing Hartmann’s procedure due to perforated sigmoidal colon diverticulitis. Among other things, he identified ASA scores higher than II as one of the negative prognostic factors for both morbidity and mortality [20]. Similarly, Anwar et al. evaluated the results of the surgical treatment of perforated colorectal cancer and determined the ASA score to be one of the negative prognostic factors in the treatment of this disease [21]. In an article in 2021, Tartaglia et al. reported a significantly higher mortality in patients treated with the open abdomen technique for abdominal sepsis, whose ASA score was higher than IV [22].

However, neither morbidity nor mortality was assessed based on the ASA score in patients with diffuse peritonitis in most of the literature data. Mortality in our peritonitis cohort was 0% in ASA I, 10.6% in ASA II, 37.8% in ASA III, and 63.6% in ASA IV, which was higher than that reported in the literature. This is certainly due to the emergent condition in patients with shock, which is common in peritonitis, yielding ASA I-IVE assessment. Similarly, the morbidity in our group was higher than in the reported data (31% for ASA I to 81.8% for ASA IV). This was again primarily caused by the lack of literature data in patients with diffuse peritonitis. However, a gradual increase in the frequency of surgical complications with increasing ASA score, similarly to MPI, is clearly apparent.

A similar, although not as exact, correlation was seen for the ECOG scoring system, which was primarily utilized to assess the overall condition of cancer patients based on their functional abilities, self-sufficiency, etc. [23]. Our group showed a gradual progression of mortality with increasing ECOG, but a slight decrease in mortality could be seen with increasing ECOG in patients evaluated as ECOG 3 and ECOG 4. A similar trend could be seen in the morbidity—there was also a decrease in the ECOG 4 group compared to ECOG 3, probably caused by subjective ECOG status evaluation, but also the group sizes—there were only four ECOG-4 patients in our group.

It seems that the use of simpler scoring systems primarily intended for other indications (ASA, ECOG, possibly qSOFA) could correlate well with the commonly used scoring systems for the prediction of mortality and morbidity in patients with diffuse peritonitis (MPI). The main benefits also include their simplicity, wide use in surgical fields, and the possibility of cooperation with other specialists such as anesthesiologists, who commonly use ASA. ASA seems to be optimal and more valid for various reasons—it is more widely used in surgical fields and is common in most workplaces. In our opinion, it assesses the patient condition better than the more subjective ECOG Performance status. However, further prospective multicentric studies will be necessary to verify the postulate.

We are aware that our approach suffers from certain limitations, which should be discussed in future studies. The first limitation was in the study design, where the data (ASA and ECOG scores) were acquired in an urgent condition of the patient in shock and hence could not be fully validated. Where ASA or ECOG was not calculated in the preoperative period, the score had to be calculated retrospectively based on the medical records, which could lead to certain disbalance between the individual patients and their score evaluation. A further limitation was the very result of the simple scoring system, which should not tempt the physician to make a simple interpretation of the patient’s prognosis based on the data thus obtained. In contrast, the simple results must be critically subjected to additional evaluation during the further treatment in order to verify the results. Our postulate could undoubtedly be conveniently verified by using multivariate analyses, which can provide additional conclusions and confirm or refute our results. The inclusion of the different types of peritonitis, with different etiology, in our system may pose another problem. However, since we attempted to create a model based on which we would be able to determine the patient’s prognosis before the surgery, we included all peritonitis cases in our cohort, regardless of the etiology. Last but not least, the monocenter nature of the study is also a disadvantage. However, it should also be noted that confining the study to a single site also had advantages (e.g., reduction in random errors due to a lower number of operating surgeons compared to a multicenter study).

## 5. Conclusions

The results of the treatment of diffuse peritonitis are still unsatisfactory, and optimal procedures are still being sought to reduce both mortality and morbidity. It is most important to identify patients who will require the most aggressive therapy possible, in order to minimize the negative consequences of the disease. There are multiple scoring systems able to predict the extent of these complications. In addition to well-established and widely used systems such as MPI, there are also scoring systems utilized in the evaluation of peritonitis rarely or to a limited extent (e.g., qSOFA). New scoring systems are still being developed to further refine the prediction of a severe development of the disease. For instance, the MPPM scoring system devised by Petersen et al. seems to be a very promising tool. However, this very efficient system requires a large volume of input data, and for this reason, we were looking for simpler systems possessing a similar predictive value. There are simple scoring systems widely used in surgical practice, but without a primary relation to diffuse peritonitis. Based on our findings, their use in patients with diffuse peritonitis correlates relatively well with established systems, as is the case within ASA score and ECOG Performance status. ASA score seems to also be particularly useful in this indication. It is a standard and widely used scoring system, both in elective and acute or emergency surgery. It is a precise system that is still used mainly by anesthesiologists, although it is relatively simple and can be easily calculated even in conditions of urgent access to patients in acute life-threatening conditions. In our group, the ASA results correlated very well with the prediction of mortality in comparison with the established scoring systems. However, a revision of the literature data also points to the need for further research of our results, preferably in a detailed multidisciplinary prospective multicentric study with a large patient cohort to ensure that the result is sufficiently representative.

## Figures and Tables

**Table 1:** Dependence of morbidity and mortality on age.

	Total Mortality										*p*
	Yes (n = 62)					No (n = 212)					
	Median	Min	Max	Mean	SD	Median	Min	Max	Mean	SD	
Age	69.0	45.0	92.0	70.1	11.4	62.0	1.5	95.0	59.5	17.2	<0.0001
	**Morbidita Celkově**										***p***
	**Yes (n = 201)**					**No (n = 73)**					
	**Median**	**Min**	**Max**	**Mean**	**SD**	**Median**	**Min**	**Max**	**Mean**	**SD**	
Age	66.0	9.0	95.0	64.4	14.9	59.0	1.5	91.0	55.1	19.3	0.001

**Table 2:** Mortality in individual groups according to qSOFA, ASA, MPI, and ECOG.

		Total Mortality				*p*
		Yes		No		
		Number	%	Number	%	
qSOFA	0	9	6.6%	128	93.4%	<0.0001
	1	25	27.5%	66	72.5%	
	2	22	59.5%	15	40.5%	
	3	6	66.7%	3	33.3%	
ASA	I	0	0%	29	100.0%	<0.0001
	II	13	10.6%	110	89.4%	
	III	42	37.8%	69	62.2%	
	IV	7	63.6%	4	36.4%	
MPI	0–20	5	8.6%	53	91.4%	<0.0001
	21–29	10	9.0%	101	91.0%	
	30 and more	47	44.8%	58	55.2%	
ECOG	0	2	3.4%	56	96.6%	<0.0001
	1	16	16.8%	79	83.2%	
	2	28	30.4%	64	69.6%	
	3	14	56.0%	11	44.0%	
	4	2	50.0%	2	50.0%	

**Table 3:** Morbidity in individual scoring systems.

		Total Morbidity				*p*
		Yes		No		
		Number	%	Number	%	
qSOFA	0	78	56.9%	59	43.1%	<0.0001
	1	81	89.0%	10	11.0%	
	2	34	91.9%	3	8.1%	
	3	8	88.9%	1	11.1%	
ASA	I	9	31.0%	20	69.0%	<0.0001
	II	89	72.4%	34	27.6%	
	III	94	84.7%	17	15.3%	
	IV	9	81.8%	2	18.2%	
MPI GROUP 1,2,3	1	25	43.1%	33	56.9%	<0.0001
	2	81	73.0%	30	27.0%	
	3	95	90.5%	10	9.5%	
ECOG	0	26	44.8%	32	55.2%	<0.0001
	1	74	77.9%	21	22.1%	
	2	76	82.6%	16	17.4%	
	3	22	88.0%	3	12.0%	
	4	3	75.0%	1	25.0%	

## Data Availability

All data presented in this study are included in this article.
